# Methods, outcomes, and costs of a 2.5 year comprehensive facility-and community-based HIV testing intervention in Bukoba Municipal Council, Tanzania, 2014-2017

**DOI:** 10.1371/journal.pone.0215654

**Published:** 2019-05-02

**Authors:** Haddi Jatou Cham, Duncan MacKellar, Haruka Maruyama, Oscar Ernest Rwabiyago, Omari Msumi, Claire Steiner, Gerald Kundi, Rachel Weber, Johnita Byrd, Chutima Suraratdecha, Tewodaj Mengistu, Eliufoo Churi, Sherri Pals, Caitlin Madevu-Matson, Geofrey Alexander, Sarah Porter, Kokuhumbya Kazaura, Deogratius Mbilinyi, Fernando Morales, Thomas Rutachunzibwa, Jessica Justman, Anath Rwebembera

**Affiliations:** 1 Division of Global HIV and TB, National Center for Global Health, U.S. Centers for Disease Control and Prevention, Atlanta, Georgia, United States of America; 2 ICAP at Columbia University, Dar es Salaam, Tanzania; 3 U.S. Centers for Disease Control and Prevention, Dar es Salaam, Tanzania; 4 U.S. Centers for Disease Control and Prevention, Yaounde, Cameroon; 5 ICF International, Atlanta, Georgia, United States of America; 6 Henry Jackson Foundation Medical Research International, Mbeya, Tanzania; 7 ICAP at Columbia University, New York, New York, United States of America; 8 Tanzania Health Promotion Support, Dar es Salaam, Tanzania; 9 United Nations Children’s Fund, Dar es Salaam, Tanzania; 10 Ministry of Health, Community Development, Gender, Elderly and Children, Bukoba, Tanzania; 11 National AIDS Control Program, Ministry of Health, Community Development, Gender, Elderly and Children, Dar es Salaam, Tanzania; AIDS Healthcare Foundation, UNITED STATES

## Abstract

To diagnose ≥90% HIV-infected residents (diagnostic coverage), the Bukoba Combination Prevention Evaluation (BCPE) implemented provider-initiated (PITC), home- (HBHTC), and venue-based (VBHTC) HIV testing and counseling (HTC) intervention in Bukoba Municipal Council, a mixed urban and rural lake zone community of 150,000 residents in Tanzania. This paper describes the methods, outcomes, and incremental costs of these HTC interventions. PITC was implemented in outpatient department clinics in all eight public and three faith-based health facilities. In clinics, lay counselors routinely screened and referred eligible patients for HIV testing conducted by HTC-dedicated healthcare workers. In all 14 wards, community teams offered HTC to eligible persons encountered at 31,293 home visits and at 79 male- and youth-frequented venues. HTC was recommended for persons who were not in HIV care or had not tested in the prior 90 days. BCPE conducted 133,695 HIV tests during the 2.5 year intervention (PITC: 88,813, 66%; HBHTC: 27,407, 21%; VBHTC: 17,475, 13%). Compared with other strategies, PITC conducted proportionally more tests among females (65%), and VBHTC conducted proportionally more tests among males (69%) and young-adults aged 15–24 years (42%). Of 5,550 (4.2% of all tests) HIV-positive tests, 4,143 (75%) clients were newly HIV diagnosed, including 1,583 males and 881 young adults aged 15–24 years. Of HIV tests conducted 3.7%, 1.8%, and 2.1% of PITC, HBHTC, and VBHTC clients, respectively, were newly HIV diagnosed; PITC accounted for 79% of all new diagnoses. Cost per test (per new diagnosis) was $4.55 ($123.66), $6.45 ($354.44), and $7.98 ($372.67) for PITC, HBHTC, and VBHTC, respectively. In a task-shifting analysis in which lay counselors replaced healthcare workers, estimated costs per test (per new diagnosis) would have been $3.06 ($83.15), $ 4.81 ($264.04), and $5.45 ($254.52), for PITC, HBHTC, and VBHTC, respectively. BCPE models reached different target groups, including men and young adults, two groups with consistently low coverage. Implementation of multiple models is likely necessary to achieve ≥90% diagnostic coverage.

## Introduction

The Joint United Nations Programme on HIV/AIDS (UNAIDS) 90-90-90 targets proposes that epidemic control will be achieved by 2030 if 90% of all people living with HIV (PLHIV) are diagnosed (diagnostic coverage), 90% of diagnosed PLHIV receive sustained antiretroviral therapy (ART), and 90% of PLHIV on ART are virally suppressed by 2020 [1]. Diagnosis of HIV infection is thus necessary for timely ART, which substantially reduces HIV-related mortality and HIV transmission risk to partners and offspring [2, 3].

Achieving 90-90-90 is particularly important in Tanzania, a country of 51.3 million persons and with an estimated 1.4 million adult PLHIV, aged 15–64 years (5.0% HIV prevalence) [4]. To increase diagnostic coverage, the Tanzania Ministry of Health, Community Development, Gender, Elderly and Children (MoHCDGEC) released comprehensive HIV testing and counseling (HTC) guidelines in 2013 for both healthcare facility and community settings. The national guidelines recommended provider-initiated HTC (PITC) for all patients seeking health care services in facilities, and home- (HBHTC) and venue-based (VBHTC) HTC in communities for persons who have limited access to or who do not regularly use health care (e.g. residents in rural areas, men, young people, and key populations) [5].

The national HTC guidelines, however, have not been fully implemented, although Tanzania has made significant progress in diagnosing and maintaining 846,257 adults and children on ART in 2016 [6]. Despite this progress, many PLHIV remain undiagnosed [4]. In 2017, only an estimated 52% of PLHIV aged 15–64 years knew their HIV positive status, and among male PLHIV, only 45% knew their HIV status [4]. In the context of limited resources, achieving 90% diagnostic coverage in Tanzania in the shortest period of time requires identifying HTC strategies that can diagnose the most persons across demographic groups at the lowest costs. Thus, knowledge of differential HTC costs and yield of new HIV-positive diagnosis (new diagnosis) when facility and community HTC strategies are implemented in accordance with national guidelines in both urban and rural communities is needed to guide service delivery.

Although studies in Tanzania and elsewhere have evaluated PITC and community-based testing strategies, [7–23] none have directly compared the relative contribution of these strategies to new diagnoses identified and their associated costs when implemented in the same geographic area and time period. Additionally, when PITC has been evaluated in outpatient department (OPD) clinics in several studies, implementation practices fell short of routine PITC as recommended by national guidelines [24–26]. Therefore, little is known about yield of new diagnosis that can be achieved through routine PITC in OPD clinics.

In a mixed urban and rural Lake-zone community of approximately 150,000 residents, the Bukoba Combination Prevention Evaluation (BCPE) implemented a community-wide HTC intervention to help achieve the UNAIDS goal of diagnosing ≥90% of HIV-infected adult residents [27]. In accordance with national HTC guidelines, BCPE comprehensively implemented PITC, HBHTC, and VBHTC throughout rural and urban wards of Bukoba Municipal Council (BMC) over a 2.5 year period, from October 2013 through March 2017.

This paper describes the methods, outcomes, and incremental costs of BCPE’s facility- and community-based HTC intervention. Outcomes reported include tests conducted; program costs and unit cost per test and per new diagnosis by HTC strategy; and the relative contribution of each strategy to all new diagnoses identified overall, and among sex, age group, and urban/rural subgroups. Findings from this paper may inform the allocation of resources for nationally recommended HTC strategies to help achieve 90% diagnostic coverage in Tanzania and similar settings.

## Methods

### Study population

**Fig 1:**
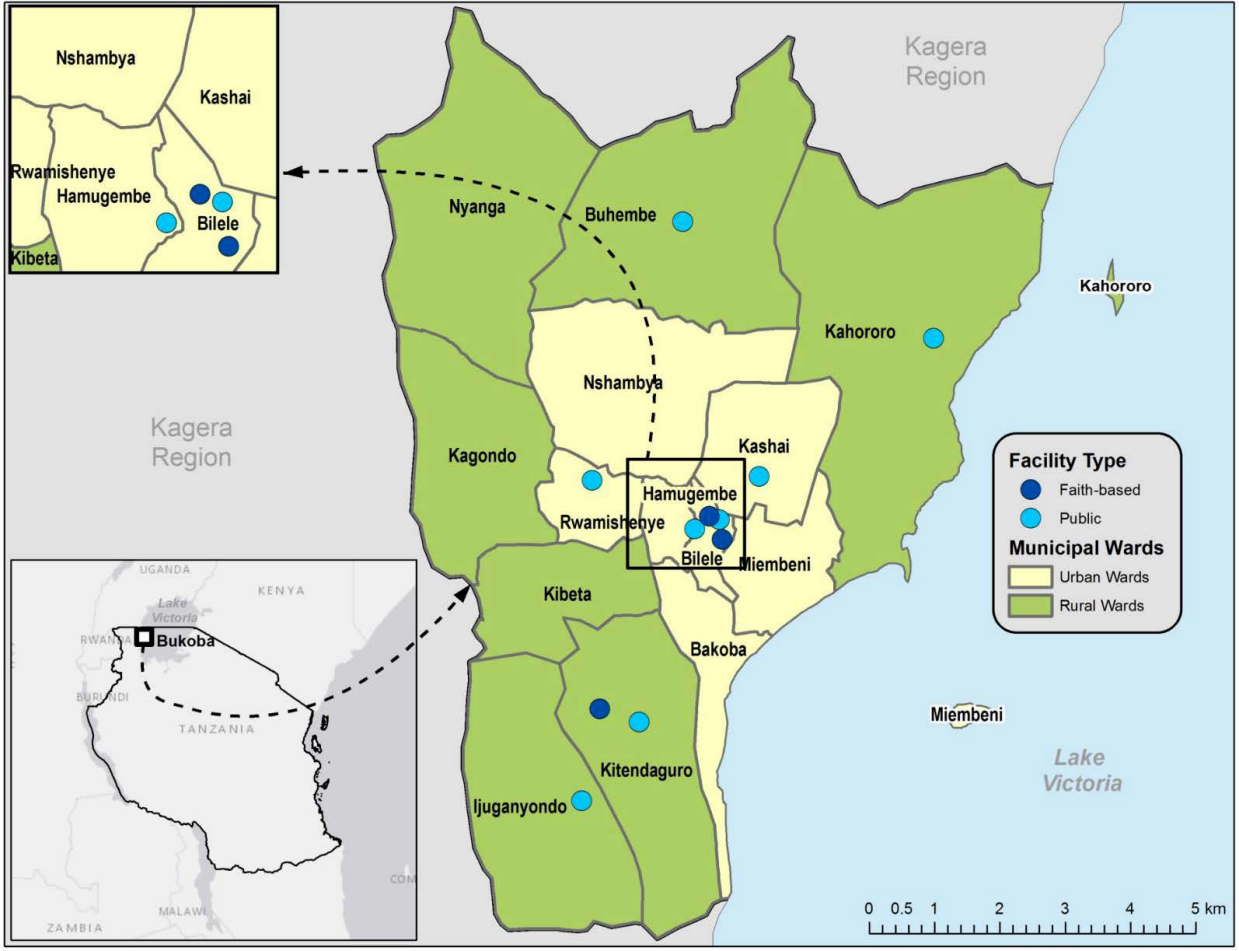
Map of Bukoba Municipal Council, Kagera Region, Tanzania. ^**a** a^Estimated population in 2017 = 150,867. Bukoba Municipal Council is a geographic district within Kagera Region. Provider initiated HIV testing and counseling was implemented in 11 faith-based and government healthcare facilities.

BMC, the capital of Kagera Region, is located on the western shore of Lake Victoria and has 14 urban and rural wards (Fig 1), which include two island communities. Approximately 73% of residents live in urban wards [28]. Fishing and agriculture are major economic industries in BMC. Residents of these islands are primarily fisherfolk, defined as fishermen and associated populations that support the fishing industry, including sex workers [29]. In 2013, the estimated HIV prevalence among adults aged 18–49 years in BMC was 9.1% and recent testing was low with only 52% of men and 68% of women reported receiving an HIV test in the past 2 years [30, 31].

### HTC procedures

All HTC strategies were implemented with a common set of pretest counseling, screening, HIV testing, and posttest counseling procedures. Pre- and post-test counseling and rapid HIV testing was conducted in accordance with Tanzania National HTC Guidelines [5]. Trained HIV-positive, lay counselors typically provided group pre-test counseling for facility- and venue-based HTC, and couples or individual pre-test counseling for HBHTC. After counseling, all clients were individually screened to assess their eligibility for HTC. For all three strategies, clients were considered eligible if they never tested for HIV, tested HIV-negative more than 90 days ago, or were previously diagnosed HIV-positive but had not received HIV care in the past 90 days (previously diagnosed, out-of-care). Ineligible clients were not denied testing if they insisted on HTC.

Eligible clients were directed to a designated healthcare worker, typically a nurse counselor, who conducted HIV testing using the national serial rapid-test algorithm. In Tanzania, HIV rapid tests must be administered by healthcare workers. In all facility- and high volume venue-based settings, the healthcare worker conducted multiple concurrent tests, typically keeping track of up to five HIV rapid tests at any time. Positive results on the first rapid test (Alere Determine HIV-1/2 or SD BIOLINE HIV-1/2) were confirmed with a second rapid test (Uni-Gold HIV). After clients received their test results from the healthcare worker, lay counselors provided individual post-test counseling. All clients who tested HIV-positive were screened with a standard form to re-assess whether they had been previously diagnosed and received HIV care in the past 90 days (previously diagnosed, in-care). Clients who reported being newly diagnosed or previously diagnosed, out-of-care were eligible for the BCPE linkage case management program, which helped them enroll in HIV care [32].

#### PITC

PITC was implemented in outpatient department (OPD) clinics in 11 health facilities, including three faith-based health centers and all eight government facilities excluding military and police clinics (Fig 1). Government facilities included one regional referral hospital, two health centers, and five dispensaries. Depending on the facility, OPDs included combined or separate primary and reproductive child health care clinics. BCPE’s PITC model aimed to fully implement national guidelines on routinely offering HTC to patients who visit the OPD. BCPE’s PITC model aimed to screen all OPD clinic patients and offer HTC to those who are eligible patients as a routine standard of care. To implement the model, staffing was increased at each clinic by at least one full-time healthcare worker and four part-time lay counselors. PITC was offered Monday through Friday from 8:00 am to 3:30 pm. Six facilities started implementing the model in October 2014 while the rest started in December 2014. Before BCPE, PITC was conducted by clinicians and was not routinely offered to OPD patients in these facilities.

#### Community-based HTC (CBHTC)

CBHTC was conducted between December 2014 and March 2017 in homes (HBHTC) and venues (VBHTC) in all 14 BMC wards with five to seven teams, each consisting of approximately four part-time lay counselors and one full-time healthcare worker. Before implementing CBHTC in a ward, the community mobilizer worked with respective community leaders to identify potential venues for VBHTC, map out neighborhoods, enumerate households for HBHTC, and sensitize community residents of upcoming CBHTC services. Before BCPE, CBHTC was not offered in BMC.

#### VBHTC

Community teams began VBHTC in December 2014 and aimed to increase HTC uptake among men and young adults. It was conducted either indoors or outdoors at high-traffic settings including community events, social venues frequented by men and young adults (e.g. sporting events, faith settings), hotspots (e.g. bars, night clubs etc.), and workplaces (e.g. markets, mechanic shops, transportation hubs etc.). To conduct HTC, teams set up pre-test counseling, screening, testing, and post-testing counseling stations in tents if venues were outdoors or in private areas or rooms if venues were indoors. Depending on the setting, teams stayed at one venue for one to three days, or rotated to different venues on a given day. In nearly all wards, VBHTC was conducted first to launch CBHTC activities followed by HBHTC. VBHTC occurred more frequently in urban wards, while it was only conducted in rural wards during well-attended community events or to begin CBHTC activities, if an appropriate venue was available.

#### HBHTC

Community teams began HBHTC in January 2015 and aimed to visit all BMC homes and screen and offer HTC to all encountered eligible persons. HTC was conducted in private rooms or areas within or near homes from 8:00 a.m. to 5:00 p.m., Monday through Saturday. Teams remained in the specified ward until all households were visited at least once. Households in which residents were not encountered were typically revisited at least once. In July 2016, HBHTC was completed on the mainland in all BMC wards. In November 2016, HBHTC was conducted on the two island communities of BMC. From November 2016 to March 2017, targeted HBHTC was conducted on the mainland but restricted to home clusters that included partners and family members of PLHIV diagnosed during VBHTC events.

### Data collection and analysis

#### HTC outcomes

Healthcare workers and lay counselors used project-specific screening forms to assess and record clients’ eligibility for HTC and MoHCDGEC HTC register to document testing outcomes. On a monthly basis, trained data clerks validated and compiled counts of HIV tests conducted and diagnostic outcomes for each HTC strategy by sex, age group, and geographic setting (urban/rural). Senior staff periodically validated aggregate monthly data against source forms for quality assurance purposes. This paper reports counts of tests conducted and proportion of tested clients newly and previously diagnosed, out-of-care by HTC strategy, sex, age group, geographic setting, and time period (calendar-year quarter). The contribution of all tests conducted and new diagnoses identified by test strategy is also reported overall, and by sex and age group. Statistical tests were not applied to compare group differences because HTC clients represent the population of interest.

#### HTC program costs

An incremental approach was used to estimate program costs and unit costs per test and per new diagnosis overall and by strategy from the provider perspective. Cost data were collected retrospectively from project accounts and interviews with staff in August 2017. Cost per test and per new diagnosis were also estimated for a task shifting model in which lay counselors replaced healthcare workers to implement these HTC strategies. Personnel, training, commodities, supplies, equipment, and travel, were calculated for each strategy. Costs for test kits used per client were calculated by multiplying unit costs of test kits (SD-BIOLINE and Uni-gold) in 2017 by the estimated number of kits used to test clients reached by each strategy. Vehicle costs were annuitized at a rate of 3% a year over an assumed expected useful life of 5 years. PITC utilized existing infrastructure and utilities at the facilities and did not incur any additional costs for these inputs. VBHTC and HBHTC did not incur any infrastructure or utility costs. All costs were collected in Tanzanian Shillings (TZS), inflated to 2017 price levels using the annual Tanzania consumer price index (CPI) ratio of 1.05, 1.11 and 1.17 for 2014, 2015 and 2016 [33], respectively, and converted to 2017 U.S. Dollars (USD) using the 2017 average market exchange rate (USD 1 = TZS 2,269.27 [34]).

### Ethical review

The BCPE study including HTC procedures and forms were approved by Institutional Review Boards of the Government of Tanzania’s National Institute for Medical Research and Columbia University, New York. The study was also reviewed according to U.S. Centers for Disease Control and Prevention (CDC) human research protection procedures and was approved as research but CDC involvement did not constitute engagement in human subject research. Per Tanzania’s national HIV testing guidelines, all persons who tested for HIV first provided verbal informed consent. Parental consent was obtained for clients aged <18 years unless they were mature minors—defined as any person below 18 years of age who is married, pregnant, sexually active, or otherwise believed to be at risk for HIV infection [5]. Before HIV test administration, healthcare workers obtained and recorded informed verbal consent from all persons on the national HTC register.

## Results

### Tests conducted by demographic characteristics and strategy

Over the 2.5 year intervention period, 138,259 facility and community clients screened eligible for HTC and 133,695 tests were conducted (Table 1). Of these tests, 42% (n = 56,304) were among males, 79% (n = 106,682) were among clients aged 15–49 years, and 75% (n = 100,580) were conducted in urban wards. PITC teams conducted 88,813 tests in 11 facilities and tested proportionally more females (65%) than HBHTC (53%) and VBHTC (31%). CBHTC teams conducted 27,407 tests during 31,293 home visits and 17,475 tests at 79 venues. HBHTC tested proportionally more children and adolescents aged <15 years (22%) and persons in rural wards (35%) than PITC (14%, 24%) and VBHTC (2%, 15%), respectively. VBHTC tested proportionally more males (69%) and persons aged 15–24 years (42%) than PITC (35%, 30%) and HBHTC (47%, 34%), respectively. The proportion of males aged 15–49 years tested through VBHTC (65%) was about twice that of HBHTC (33%) and PITC (25%).

**Table 1:** HIV tests conducted, by demographic characteristics and HIV testing strategy, Bukoba Combination Prevention Evaluation, Tanzania, 2014–2017^a^.

	Total		PITC			HBHTC			VBHTC		
	n	%^b^	n	%^b^	%^c^	n	%^b^	%^c^	n	%^b^	%^c^
**Total**	133,695	100	88,813	100	66	27,407	100	21	17,475	100	13
**Sex**											
Male	56,304	42	31,329	35	56	12,917	47	23	12,058	69	21
Female	77,391	58	57,484	65	74	14,490	53	19	5,417	31	7
**Age group**											
<15	19,204	14	12,785	14	67	6,146	22	32	273	2	1
15–24	43,247	32	26,644	30	62	9,337	34	22	7,266	42	17
25–49	63,435	47	44,122	50	70	10,257	37	16	9,056	52	14
>49	7,809	6	5,262	6	67	1,667	6	21	880	5	11
**Male**											
<15	9,425	7	6,260	7	66	3,027	11	32	138	1	1
15–24	16,888	13	7,512	8	44	4,342	16	26	5,034	29	30
25–49	26,041	19	15,034	17	58	4,778	17	18	6,229	36	24
>49	3,950	3	2,523	3	64	770	3	19	657	4	17
**Female**											
<15	9,779	7	6,525	7	67	3,119	11	32	135	1	1
15–24	26,359	20	19,132	22	73	4,995	18	19	2,232	13	8
25–49	37,394	28	29,088	33	78	5,479	20	15	2,827	16	8
>49	3,859	3	2,739	3	71	897	3	23	223	1	6
**Setting**^**d**^											
Urban	100,580	75	67,769	76	67	17,920	65	18	14,891	85	15
Rural	33,115	25	21,044	24	64	9,487	35	29	2,584	15	8

HIV, human immunodeficiency virus; PITC, provider initiated testing and counseling; HBHTC, home-based HIV testing and counseling; VBHTC, venue-based HIV testing and counseling.

^a^ PITC was conducted in outpatient department clinics in government and faith-based facilities. HBHTC was offered to encountered residents within or near their homes. VBHTC was conducted either indoors or outdoors at high-traffic settings including community events, social venues frequented by men and young adults (e.g., sporting events and faith settings), hotspots (e.g., bars and night clubs), and workplaces (e.g., markets and transportation hubs).

^b^ Of tests conducted, overall and by strategy (column %).

^c^ Of total tests conducted (row %).

^d^ Urban (n = 7) and rural (n = 7) wards.

### Contribution of strategies to all tests conducted

PITC, HBHTC, and VBHTC accounted for 66%, 21%, and 13%, respectively, of 133,695 tests conducted (Table 1). PITC accounted for the majority of tests (≥ 56%) conducted among all sex, age, and geographic subgroups, except males 15–24 years (44%). Of 16,888 males aged 15–24 years who were tested, VBHTC and HBHTC accounted for 30% and 26%, respectively. Of the two CBHTC strategies, HBHTC accounted for proportionally more tests than VBHTC among all demographic groups except among males aged 15–49 years (Table 1). On the two islands of BMC, HBHTC accounted for 36% and VBHTC contributed 64% of 484 tests conducted.

### Trends in tests conducted

**Fig 2:**
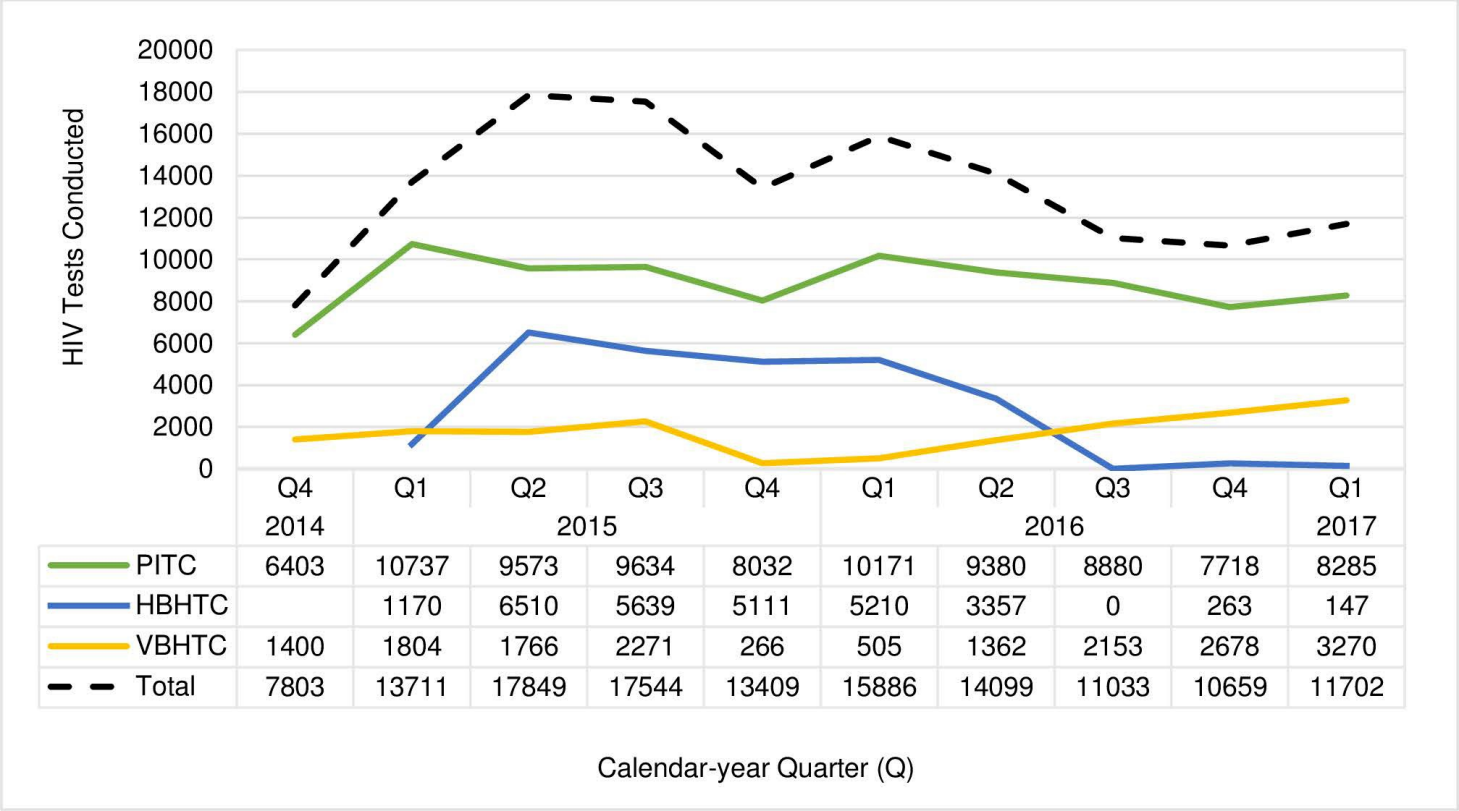
HIV tests conducted by calendar-year quarter and HIV testing strategy, Bukoba Combination Prevention Evaluation, Bukoba Municipal Council, Tanzania, 2014-2017. ^**a**^ PITC, provider initiated testing and counseling; HBHTC, home-based HIV testing and counseling; VBHTC, venue-based HIV testing and counseling. ^a^ PITC began in Q4 2014 in seven of 11 health facilities and was implemented in all 11 facilities during Q1 2015 –Q1 2017. HBHTC teams visited all Bukoba Municipal Council (BMC) households on the mainland and offered HIV testing to encountered persons during Q1 2015 –Q2 2016. HBHTC was not conducted during Q3 2016. In Q4 2016, HBHTC teams visited all households and offered HIV testing on two BMC islands. In Q1 2017, HBHTC was restricted to home clusters on the mainland that included partners and family members of persons diagnosed during VBHTC events. VBHTC was conducted on the mainland from Q4 2014 –Q1 2017 and on the islands in Q4 2016. VBHTC was conducted primarily in urban wards during Q4 2014 –Q3 2015, rural wards during Q4 2015 –Q1 2016, and urban wards Q2 2016 –Q1 2017.

During the intervention period (quarter [Q] 4 2014 through Q1 2017), an average of 13,365 tests were conducted per quarter (Fig 2). After the initial scale up to all 11 facilities in Q4 2014, the average number of PITC tests remained high and stable with an average of 9,157 tests per quarter. From Q1 2015 through Q2 2016, HBHTC teams visited households on the mainland and conducted an average of 4,500 tests per quarter. VBHTC teams conducted an average of 1,810 tests per quarter between Q4 2014 through Q3 2015, before declining to 386 per quarter between Q4 2015 and Q1 2016 when community teams were primarily in rural wards. VBHTC increased steadily from Q2 2016 through Q1 2017 with an average of 2,366 tests per quarter as teams shifted back to urban areas. In Q4 2016 and Q1 2017, 410 tests were conducted at homes on the two islands and clusters of homes on the mainland to test partners and family members of consenting clients diagnosed during VBHTC events.

### New HIV diagnoses identified by demographic characteristics and strategy

Of 133,695 tests conducted, 5,550 (4.2%) were HIV-positive. Of HIV-positive tests, 4,143 (75%) clients were newly diagnosed, and 588 (11%) and 891 (14%) had been previously diagnosed, and were out-of-care and in-care, respectively. Over the 2.5 year intervention period, 1,583 males (2.8%), 2,560 females (3.3%), and 3,705 persons aged 15–49 years (3.5%) were newly diagnosed (Table 2). The proportion of PITC clients newly diagnosed (3.7%) was higher than HBHTC (1.8%) and VBHTC (2.1%) overall and in most demographic subgroups (Table 2). Of 29,991 tests among adult males >24 years of age, the proportion of PITC clients newly diagnosed (6.0%) was 1.9 and 2.7 times higher than HBHTC (3.1%) and VBHTC (2.2%), respectively. Of 41,253 tests among adult females >24 years of age, the proportion of PITC clients newly diagnosed (4.6%) was 1.6 and 1.4 times higher than HBHTC (2.9%) and VBHTC (3.3%), respectively.

**Table 2:** New HIV diagnoses, by demographic characteristics and HIV testing strategy, Bukoba Combination Prevention Evaluation, Bukoba Municipal Council, Tanzania, 2014–2017^a^.

	Total		PITC			HBHTC			VBHTC		
	n	%^b^	n	%^b^	%^c^	n	%^b^	%^c^	n	%^b^	%^c^
**Total**	4,143	3.1	3,270	3.7	78.9	499	1.8	12.0	374	2.1	9.0
**Sex**											
Male	1,583	2.8	1,182	3.8	74.7	203	1.6	12.8	198	1.6	12.5
Female	2,560	3.3	2,088	3.6	81.6	296	2.0	11.6	176	3.2	6.9
**Age group**											
<15	137	0.7	119	0.9	86.9	16	0.3	11.7	2	0.7	1.5
15–24	881	2.0	638	2.4	72.4	125	1.3	14.2	118	1.6	13.4
25–49	2,824	4.5	2,257	5.1	79.9	329	3.2	11.7	238	2.6	8.4
>49	301	3.9	256	4.9	85.0	29	1.7	9.6	16	1.8	5.3
**Male**											
<15	51	0.5	44	0.7	86.3	7	0.2	13.7	0	0.0	0.0
15–24	146	0.9	80	1.1	54.8	22	0.5	15.1	44	0.9	30.1
25–49	1,226	4.7	925	6.2	75.4	157	3.3	12.8	144	2.3	11.7
>49	160	4.1	133	5.3	83.1	17	2.2	10.6	10	1.5	6.3
**Female**											
<15	86	0.9	75	1.1	87.2	9	0.3	10.5	2	1.5	2.3
15–24	735	2.8	558	2.9	75.9	103	2.1	14.0	74	3.3	10.1
25–49	1,598	4.3	1,332	4.6	83.4	172	3.1	10.8	94	3.3	5.9
>49	141	3.7	123	4.5	87.2	12	1.3	8.5	6	2.7	4.3
**Setting**^**d**^											
Urban	3,337	3.3	2,698	4.0	80.9	334	1.9	10.0	305	2.0	9.1
Rural	806	2.4	572	2.7	71.0	165	1.7	20.5	69	2.7	8.6

HIV, human immunodeficiency virus; PITC, provider initiated testing and counseling; HBHTC, home-based HIV testing and counseling; VBHTC, venue-based HIV testing and counseling.

^a^ All HIV-positive clients were screened to assess their new or prior diagnostic status. HIV-positive clients who reported having never previously tested HIV-positive were defined as newly HIV diagnosed. PITC was conducted in outpatient department clinics in government and faith-based facilities. HBHTC was offered to encountered residents within or near their homes. VBHTC was conducted either indoors or outdoors at high-traffic settings including community events, social venues frequented by men and young adults (e.g., sporting events and faith settings), hotspots (e.g., bars and night clubs), and workplaces (e.g., markets and transportation hubs).

^b^ New HIV diagnoses divided by tests conducted (Table 1).

^c^ Of total new HIV diagnoses.

^d^ Urban (n = 7) and rural (n = 7) wards where testing was conducted.

### Contribution of strategies to all new HIV diagnoses identified

PITC identified 79% of all new diagnoses and >70% of new diagnoses across all demographic groups, with the exception of young adult males aged 15–24 years. PITC, HBHTC, and VBHTC identified 55%, 15%, and 30% of new diagnoses among young adult males, respectively (Table 2). HBHTC (12% of all new diagnoses) and VBHTC (9% of all new diagnoses) identified 21% and 9% of new diagnoses in rural wards (Table 2), respectively, and 32% and 68% of 47 new diagnoses in the two islands of BMC. PITC also identified 85% (497/588) of previously diagnosed, out-of-care clients.

### Trends in new HIV diagnoses

**Fig 3:**
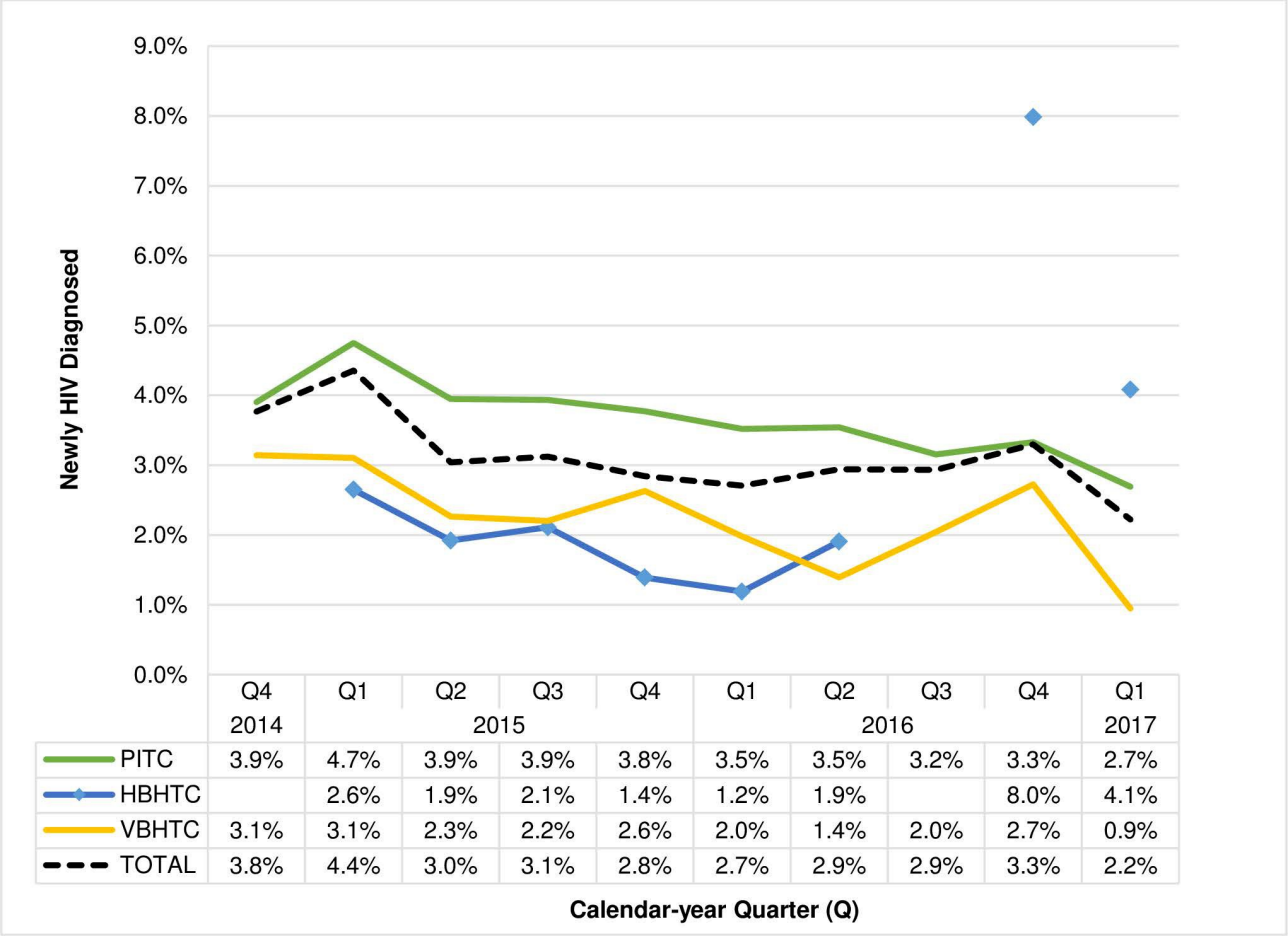
Proportion of clients newly HIV diagnosed by calendar-year quarter and HIV testing strategy, Bukoba Combination Prevention Evaluation, Bukoba Municipal Council, Tanzania, 2014-2017. ^**a**^ HIV, human immunodeficiency virus; PITC, provider initiated testing and counseling; HBHTC, home-based HIV testing and counseling; VBHTC, venue-based HIV testing and counseling. ^a^All HIV-positive clients were screened to assess their new or prior diagnostic status. HIV-positive clients who reported having never previously tested HIV-positive were defined as newly HIV diagnosed. PITC began in Q4 2014 in seven of 11 health facilities and was implemented in all 11 facilities during Q1 2015 –Q1 2017. HBHTC teams visited all Bukoba Municipal Council (BMC) households on the mainland and offered HIV testing to encountered persons during Q1 2015 –Q2 2016. HBHTC was not conducted during Q3 2016. In Q4 2016, HBHTC teams visited all homes and offered HIV testing on two BMC islands. In Q1 2017, HBHTC was restricted to home clusters on the mainland that included partners and family members of persons diagnosed during VBHTC events. VBHTC was conducted on the mainland from Q4 2014 –Q1 2017 and on the islands in Q4 2016. VBHTC was conducted primarily in urban wards during Q4 2014 –Q3 2015, rural wards during Q4 2015 –Q1 2016, and urban wards Q2 2016 –Q1 2017. Proportion newly diagnosed was calculated by dividing counts of newly diagnosed clients by total HIV tests conducted for that quarter (Fig 2).

Of HIV tests conducted, the proportion of new diagnoses was highest in the first two quarters of implementation and then steadily declined overall and for all strategies on the mainland (Fig 3). Of HTC conducted on the mainland, the proportion of new diagnoses was highest among PITC clients, followed by VBHTC and HBHTC. In Q4 2016, when CBHTC was implemented in fisherfolk communities on the two islands and on the mainland, the yield of new diagnosis was 2.7% (73/2678) for VBHTC and 8.0% (21/263) for HBHTC. Specifically, the yield of new diagnoses from VBHTC and HBHTC clients on the two islands was 10.3% (32/312) and 8.7% (15/172), respectively. While on the mainland, yield of new diagnosis from VBHTC and targeted HBHTC of household clusters of partners and family members of HIV-positive VBHTC clients was 1.7% (41/2366) and 6.6% (6/91), respectively, during this time period. In Q1 2017, VBHTC and targeted HBHTC continued on the mainland and the yield of new diagnosis among those tested was 0.9% (31/3270) and 4.1% (6/147), respectively.

**Fig 4:**
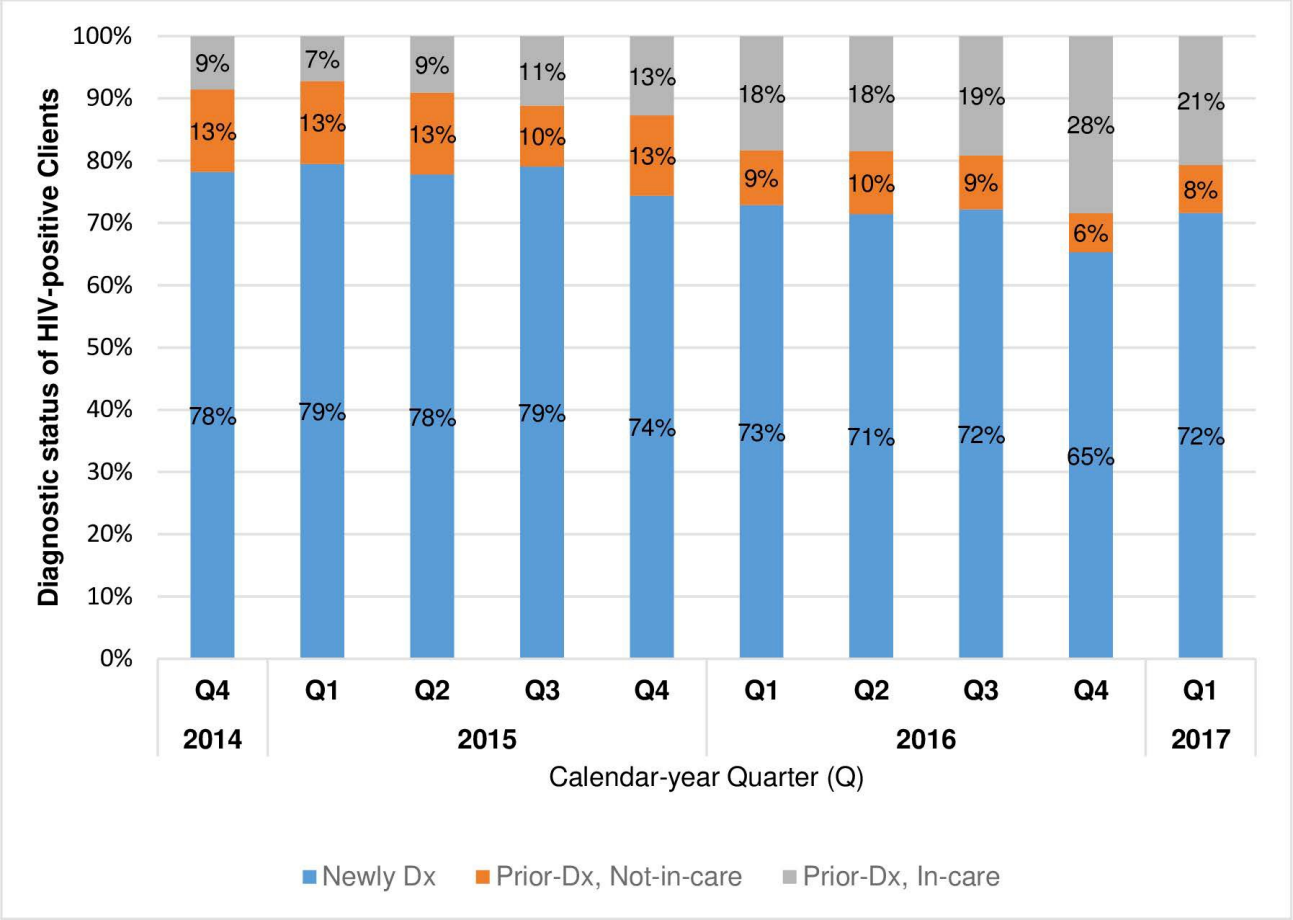
Distribution of diagnostic status of 5,550 HIV-positive clients, by calendar-year quarter, Bukoba Combination Prevention Evaluation, Bukoba Municipal Council, Tanzania, 2014-2017. ^**a**^ HIV, human immunodeficiency virus; Newly Dx, newly HIV diagnosed; Prior-Dx, Not-in-care, previously HIV diagnosed, has not received HIV care in the past 90 days; Prior-Dx, In-care, previously HIV diagnosed, has received HIV-care within the past 90 days. ^a^All HIV-positive clients were screened to assess their new or prior diagnostic status. HIV-positive clients who reported having never previously tested HIV-positive were defined as newly HIV diagnosed. Range of HIV-positive clients by calendar-year quarter = 363 (Q1 2017)– 751 (Q1 2015).

Of HIV-positive tests, the proportion of clients who were newly and previously-diagnosed, out-of-care decreased slightly over time from 78% (294/376) and 13% (50/376) in Q4 2014, respectively, to 72% (260/363) and 8% (28/363) in Q1 2017, respectively. The proportion of previously diagnosed, in-care clients increased from 9% (32/376) of positive tests in Q4 2014 to 21% (75/363) in Q1 2017 (Fig 4).

### HTC program costs

The estimated incremental costs of implementing the three HTC strategies for 2.5 years was $720,607.67. Personnel costs accounted for 57%, 46%, and 51% of PITC, HBHTC, and VBHTC costs, respectively (Table 3). The second largest cost category was test kits, which were 31%, 21% and 17% of PITC, HBHTC, and VBHTC costs, respectively. The average cost per HIV test (per new diagnosis) was $4.55 ($123.66), $6.45 ($354.44), and $7.98 ($372.67) for PITC, HBHTC, and VBHTC, respectively. In a task-shifting model with trained lay counselors assuming healthcare worker responsibilities for providing HTC, estimated cost per test (per new diagnosis) would have been $3.06 ($83.15), $4.81 ($264.04), and $5.45 ($254.42) for PITC, HBHTC, and VBHTC, respectively.

**Table 3:** Costs by HIV testing strategy, Bukoba Combination Prevention Evaluation, Bukoba Municipal Council, Tanzania, 2014–2017^a^.

	Costs			
	Total	PITC	HBHTC	VBHTC
Total Costs	$720,607.67	$404,364.89	$176,865.66	$139,377.12
Personnel^b^	$381,955.31	$229,426.29	$81,900.16	$70,628.87
Training	$35,547.29	$23,844.98	$5,851.15	$5,851.15
Test kits	$184,887.26	$123,566.90	$37,407.98	$23,912.38
Other Commodities and Supplies^c^	$59,426.36	$26,227.56	$16,599.40	$16,599.40
Equipment^d^	$4,365.25	$1,299.16	$818.47	$2,247.63
Travel^e^	$54,426.20	$0.00	$34,288.50	$20,137.69
Cost per test	$5.39	$4.55	$6.45	$7.98
Cost per new HIV diagnosis	$173.93	$123.66	$354.44	$372.67

HIV, Human immunodeficiency virus; PITC, provider initiated testing and counseling; HBHTC, home-based HIV testing and counseling; VBHTC, venue-based HIV testing and counseling

^a^All costs were collected in Tanzanian Shillings (TZS), inflated to 2017 price levels using the annual Tanzania consumer price index (CPI) ratio of 1.05, 1.11 and 1.17 for 2014, 2015 and 2016, respectively, and converted to 2017 U.S. Dollars (USD) using the 2017 average market exchange rate (USD 1 = TZS 2,269.27).

^b^ Lay counselors, nurses, and drivers (for VBHTC and HBHTC only).

^c^ Stationary, printing; medical supplies (gloves, paper towels, etc.), and staff supplies (e.g. backpacks, rain boots etc.)

^d^ Storage shelves for forms; computers; printers; and tents, benches, and chairs for VBHTC.

^e^ Vehicles, fuel consumption and mileage for HBHTC and VBHTC.

## Discussion

Over a 2.5 year intervention period, BCPE comprehensively implemented facility- and community-based HTC throughout Bukoba Municipal Council in accordance with Tanzania national testing guidelines using a standard screening method, eligibility criteria, and testing algorithm. In 11 health facilities, during 31,000 home visits, and at 79 venues, BCPE conducted over 133,000 tests and newly diagnosed 4,143 PLHIV, of whom 1,583 and 881 were males and young adults aged 15–24 years, two groups with consistently low diagnostic coverage [35]. An additional 588 PLHIV who had been previously diagnosed but were currently out-of-care were also identified. Compared to the two CBHTC strategies, facility-based PITC in OPD clinics had the lowest cost per test and new diagnosis. PITC also achieved a higher absolute number and yield of new HIV diagnoses overall, and for nearly all demographic groups. Notably, among males aged >24 years, facility-based PITC had approximately twice the yield of new HIV diagnoses compared with community-based strategies. Home- and venue-based strategies, however, tested proportionally more males and young adults aged 15–24 years, and HBHTC tested proportionally more rural residents.

Since recommended by World Health Organization in 2007, routine PITC has been successfully implemented in many antenatal and tuberculosis clinics, but in few OPD clinics in sub-Saharan Africa, underscoring the need to evaluate barriers and costs for providing PITC as recommended by national guidelines [24, 36–38]. High patient volume combined with inadequate funding, staff, and space in overburdened health facilities, and concerns about delaying patient visits are frequently reported barriers to routine PITC [24, 39]. However, when operationalized to address these barriers, PITC has achieved high HTC coverage and yield in clinical settings [22, 24, 40–42]. BCPE substantially increased PITC in OPD clinics by integrating services within the clinic flow, employing dedicated HTC personnel, and running multiple concurrent tests which substantially reduced burden on OPD staff and allowed patients to test without substantially delaying their visit.

Underscoring the effectiveness of PITC reported elsewhere, in our evaluation, PITC accounted for >75% of all new HIV diagnoses among young women aged 15–24 years and >82% of all new diagnoses among women of all other ages [22, 24, 37, 41, 42]. Although VBHTC and HBHTC tested proportionally more males and young adults than PITC, PITC still accounted for 75% of all new HIV diagnoses among males and 55% of young adults aged 15–24 years. While PITC alone is insufficient to achieve diagnostic coverage in the general population, our findings highlight the importance of capitalizing on every health encounter, particularly with men who often have undiagnosed advanced HIV disease, to ensure PLHIV who seek healthcare services are tested, diagnosed, and initiated on ART [43, 44].

BCPE PITC costs per test ($4.55) were lower than reported costs from other studies ($6.83-$13.73 per test in 2017 USD) [7, 23, 45–47]. The relatively lower per-test cost may be attributable, in part, to excluding costs for infrastructure and utilities, while comparative studies included these costs. Data from an HTC costing study in Tanzania found that infrastructure and utility accounted for about 24% of the total PITC costs. Adjusting for these costs, the cost per test ($5.65) would still be less than half of the unit cost ($13.73) reported from that same study [23]. Conducting multiple concurrent rapid tests in high-volume clinics may lead to economies of scale, thus lowering the cost per test. To our knowledge, our paper is the first to report cost per new HIV diagnosis for these three HTC strategies in Tanzania. Cost per new diagnosis will be an important indicator for monitoring performance of HTC strategies, especially as countries strive to achieve >90% diagnostic coverage and epidemic control with limited resources. Studies conducted in Tanzania and elsewhere in sub-Saharan Africa have reported cost per HIV-positive test for PITC ($20.60-$92.90) but not cost per new HIV diagnosis [7, 23, 45–47]. Our cost per new diagnosis for PITC ($123.66), was higher than the cost per HIV-positive test ($92.90) reported in another Tanzania study [23]. The number of new diagnosis, however, is usually lower than the total number of HIV-positive test results (sum of new and prior diagnoses). Notably, if we could have shifted HTC tasks from healthcare workers to lay counselors, our PITC cost per new HIV diagnosis would be $83.15. Lay counselors, compensated at much lower levels than nurses and other healthcare workers, are permitted to conduct rapid HIV tests in other countries [23, 45, 47]. Prior research demonstrates that with more training rapid HIV tests conducted by lay providers is accurate and of equal quality to testing by laboratory staff and health-care workers [48–50].

Although PITC dominated CBHTC strategies in identifying new HIV diagnoses, CBHTC is critical for testing persons who have limited access to or who do not regularly access health care. In rural settings with limited or remote facility-based services, door-to-door HBHTC or HTC campaigns can substantially increase testing and diagnostic coverage. Prior systematic reviews have also shown that community-based strategies identified HIV-positive individuals at higher CD4 counts and thus earlier in the course of their HIV disease than HIV-positive clients identified through facility-based HTC [38, 51]. In high prevalence settings, the cost-effectiveness of CBHTC compared with PITC might improve considerably. During the last 6 months of our intervention when HBHTC was conducted on two Lake Victoria islands (neither of which had health facilities) and restricted to home clusters that included partners and family members of persons diagnosed during VBHTC events, the quarterly yield of new HIV diagnoses through HBHTC exceeded that of PITC. VBHTC also achieved a high yield of new diagnoses when implemented in the islands. A Ugandan study that evaluated HBHTC and VBHTC among fisherfolk on Lake Victoria islands reported a similar yield of new HIV diagnoses (10%, 9.6%) as our study (8.7%, 10.3%) [29].

In addition to rural residents, men and young adults are less likely than women and older adults, respectively, to access healthcare services and test for HIV [18, 37, 52, 53]. Our VBHTC model tested over 12,000 men, including over 5,000 males aged 15–24 years. Similar to prior studies, the success of BCPE’s VBHTC model in reaching men is attributable to its targeted strategy of offering HTC at locations frequented by these subgroups or their known workplaces [18, 54]. HBHTC and VBHTC may have reached even more men and young adults if it were offered on Sundays when these subgroups were more likely to be at home or involved multiple rounds of testing in a given ward or community like several combination prevention trials [16, 55–57].

Our costs per HBHTC ($6.45) and VBHTC ($7.98) test were lower than those reported in most studies from sub-Saharan Africa (HBHTC $4.51—$21.96; VBHTC $12.96—$22.35 in USD 2017) [7, 11, 18, 20, 23, 58, 59]. Our cost per new diagnosis for HBHTC ($354.44) and VBHTC ($372.67) was slightly higher and lower than those reported by Parker and colleagues in Swaziland (2015) (HBHTC, $319.49; VBHTC, $505.77 in 2017 USD), the only CBHTC study we could find that reported cost per new HIV diagnosis [18]. Our lower VBHTC costs could be credited to primarily implementing this strategy in urban wards and during popular community events to ensure reasonably high demand for HTC to achieve greater economy of scale. In Swaziland, VBHTC was implemented in rural areas and most likely had fewer tests per event yet staffing and transportation costs remained the same across events. It is worth noting that in a task shifting context where lay counselors replaced healthcare workers, BCPE’s cost per new diagnosis would be reduced by 26–32% for each CBHTC strategy. Specifically, our cost per new diagnosis would $264.04 and $254.52 for HBHTC and VBHTC, respectively.

As reported elsewhere, our findings suggest that a combination of facility- and community-based HTC strategies will probably be necessary for countries to achieve 90% diagnostic coverage in all geographic settings and subgroups [38, 51]. In urban and mixed urban and rural communities in which both facility- and community-based HTC strategies can be implemented, however, limited data has been available to inform the prioritization of finite HTC resources for the general population. Although many studies have evaluated comprehensive door-to-door HBHTC in sub-Saharan Africa, we found only one that did so in combination with PITC in urban settings [8]. In Nairobi, Kenya, Muhula and colleagues compared facility-based PITC and client initiated testing with CBHTC; however, the relative contribution of testing strategies to new diagnoses identified and associated costs were not reported [8].

Because PITC costs per new diagnosis were approximately one-third lower than CBHTC, our findings suggest that PITC should reasonably be optimized first at high-volume OPDs and potentially other clinics in healthcare facilities, before resources are committed to CBHTC strategies for the general population. Targeted community-based HTC strategies are also needed in areas with limited or remote health facilities, to test partners and biological children of PLHIV, and to serve key and vulnerable populations who are unlikely to go to a health facility, especially when they are asymptomatic [37]. Scaling up PITC first in all clinical settings for general population HTC programming, will help ensure undiagnosed PLHIV seeking care do not leave healthcare settings without being diagnosed and linked to HIV care.

BCPE used a very inclusive eligibility criteria in accordance with national testing guidelines with the goal of diagnosing as many PLHIV as possible during the 2.5 year intervention. Yield of new HIV diagnoses achieved through facility- and community-based strategies could have been higher had we employed a more restrictive HTC eligibility criteria. However, at the time of our study, a highly sensitive and specific screening form was not available and not authorized for use per national testing guidelines. To improve the cost-effectiveness of HTC strategies, it is important for programs to assess and implement suitable HTC eligibility screening forms. Screening for symptoms of sexually transmitted infections and TB, for example, might improve yield considerably and reduce cost per new HIV diagnosis [37, 60]. Integrating testing of sexual partners and biological children of PLHIV, which has been shown to achieve 23%-64% and 4%-12.2% yield of HIV diagnoses, respectively, could also increase the cost-effectiveness of CBHTC programs [61–71].

Supporting prior reports that many PLHIV retest for HIV after diagnosis, BCPE staff used a standard form to identify 1,407 testers who had been previously diagnosed, of whom 819 were currently in HIV care [72, 73]. Thus, routine assessment of prior diagnostic and HIV-care status of all clients who test HIV-positive is critical, and may be particularly important as countries approach diagnostic coverage among PLHIV. In our 2.5 year intervention, previously diagnosed, in-care clients accounted for a steadily increasing proportion of all HIV positive tests. Notably, in the last 6 months of our intervention, more than 1 in 5 clients who tested HIV-positive reported already being in HIV care. Not excluding previously diagnosed clients from national reporting systems may lead to considerable overestimation of new HIV diagnoses, and potentially underestimation of linkage-to-care and ART initiation and coverage rates.

Our methods and findings have several important limitations. Some HTC test and diagnostic-status data may not have been accurately recorded on national registers and study forms, or compiled and transcribed accurately on monthly reporting forms. However, comprehensive quality-assurance procedures were employed throughout the intervention, and senior-investigator audits found few discrepancies between source forms (including patient medical records), registers, and monthly reporting forms. We are also unable to report the number of residents who tested and who were newly diagnosed during the 2.5 year intervention because tests conducted do not represent unique individuals and we did not collect information on BMC resident status. Another limitation is that prior diagnostic and HIV-care status were self-reported and thus subject to social-desirability bias. Although all clients were screened with standard instruments twice by HIV-positive, peer lay counselors (once before and after testing), some clients may have withheld their prior diagnosis. To minimize this bias, lay counselors tried to establish rapport with clients during posttest counseling and assure clients that their information would be kept confidential. Although testing of partners and biological children of PLHIV were conducted through BCPE and at HIV care and treatment clinics, the national register did not measure index-client testing. Index-client testing outcomes, however, were tracked throughout the intervention and reported as part of BCPE linkage case management [32]. Although an incremental approach was used to estimate program costs, HBHTC and VBHTC costs are considered equivalent to full costs since these strategies were not implemented in BMC prior to BCPE. Finally, we are unable to report testing outcomes for key and vulnerable populations (e.g., sex workers) because the national register did not measure membership in these groups.

Despite these limitations, BCPE PITC, VBHTC, and HBHTC models are promising strategies that may help communities achieve 90% HIV diagnostic coverage among all PLHIV. The BCPE models reached different target groups, including men and young adults, two populations with consistently low diagnostic coverage. In 2017, the Tanzania MoHCDGEC identified the BCPE PITC as a new service delivery model [74]. Through September 2018, three CDC-supported implementing partners have delivered the PITC model in 208 health facilities in 11 regions. In 2019, the United States President’s Emergency Plan for AIDS Relief is supporting the national scale up of the PITC model in all regions. BCPE facility and community-based HTC strategies might be considered in other contexts in which improvement in HTC coverage is needed.

## Supporting information

S1 Tables BCPE HTC outcomes data. (XLSX)

S2 Tables BCPE HTC cost data. (XLSX)
